# Acceptability and Preferences of Long‐Acting Injectable Pre‐Exposure Prophylaxis and Antiretroviral Therapy Among Men Who Have Sex With Men and People Who Inject Drugs in India: Insights for Future Implementation

**DOI:** 10.1002/jia2.70114

**Published:** 2026-04-29

**Authors:** Allison M. McFall, Talia A. Loeb, Jiban J. Baishya, Ashwini Kedar, Archit Sinha, A. K. Srikrishnan, Sunil S. Solomon, Gregory M. Lucas, Shruti H. Mehta

**Affiliations:** ^1^ Department of Epidemiology Johns Hopkins University Bloomberg School of Public Health Baltimore Maryland USA; ^2^ Division of Infectious Diseases Johns Hopkins University School of Medicine Baltimore Maryland USA; ^3^ Y.R. Gaitonde Centre for AIDS Research and Education (YRGCARE) Chennai India

**Keywords:** acceptability, antiretroviral therapy, India, long‐acting injectable, pre‐exposure prophylaxis, preferences

## Abstract

**Introduction:**

Long‐acting injectable (LAI) antiretroviral therapy (ART) and pre‐exposure prophylaxis (PrEP) have significant potential to impact the HIV epidemic, but there is little data on the acceptability of these newer technologies among people who inject drugs (PWID) and men who have sex with men (MSM) in low‐resource settings. We examined acceptability and preferences of LAI ART and PrEP among community‐based samples of PWID and MSM in India.

**Methods:**

We conducted a cross‐sectional survey of PWID and MSM in eight Indian cities (November 2022−May 2024) using respondent‐driven sampling. Participants completed a survey including socio‐demographics, substance use, risk behaviours, HIV testing/care history, acceptability of LAI ART and knowledge, acceptability, and preferences of different PrEP modalities (i.e. daily oral, monthly oral, LAI and implant). We assessed correlates of acceptability using Poisson regression models. To understand PrEP preferences, we used a modified Borda count method—a rank voting procedure.

**Results:**

Overall, 2249 MSM and 4499 PWID (98% male) were recruited. Among those previously diagnosed with HIV, 89% (MSM) and 75% (PWID) reported a very good chance they would use LAI ART. MSM experiencing unstable housing and PWID virally suppressed were more willing to use LAI ART. Twenty percent and five percent of MSM and PWID, respectively, had ever heard of PrEP. Among those without an HIV diagnosis, 77% (MSM) and 62% (PWID) reported a very good chance they would use LAI PrEP. MSM with more sexual partners and sexually transmitted infection symptoms and PWID who had heard of PrEP were more willing to use LAI PrEP. Among MSM interested in PrEP, monthly oral pills were most preferred, followed by LAI, daily oral pills and then implant. Among PWID, monthly oral pills were most preferred, followed by daily oral pills, LAI and then implant.

**Conclusions:**

MSM and PWID in India were open and interested in LAI ART and PrEP. Once these become available, programmes with thoughtful community outreach and education, alongside flexible delivery models, will be critical to success. For PrEP, continued investment in the development of extended‐duration oral formulations is warranted and valuable in order to provide a variety of HIV prevention choices.

## 1 Introduction

Long‐acting technologies for HIV treatment and prevention hold significant potential to impact the HIV epidemic globally, as adherence to a daily pill remains challenging for many due to multilevel factors [1, 2, 3, 4, 5]. Long‐acting injectable (LAI) modalities are delivered more conveniently through injections every 1–6 months, decreasing medication and time burden and providing a more confidential way to take medications, encouraging long‐term adherence [1, 6]. Recent trials have demonstrated that LAI formulations are highly effective for viral suppression among people living with HIV (PWH) [7] and preventing HIV acquisition among groups disproportionately affected by HIV [8, 9, 10, 11].

India has the second largest number of PWH globally [12], where people who inject drugs (PWID), men who have sex with men (MSM) and other key populations are disproportionately affected [13]. Among MSM and PWID in India, HIV incidence remains persistently high [14, 15], and those living with HIV have low levels of antiretroviral therapy (ART) use and struggle to achieve and maintain viral suppression [16, 17]. Expanded options for HIV treatment and prevention, such as long‐acting technologies, are needed. However, there is sparse data on the acceptability of these newer technologies among MSM and PWID in a low‐ and middle‐income country such as India. LAI ART and pre‐exposure prophylaxis (PrEP) are not yet approved for use in India, but licensing and regulatory preparations are in process. Thus, our objective was to examine the acceptability and preferences of LAI ART and PrEP among community‐based samples of PWID and MSM in India.

## 2 Methods

### 2.1 Study Design and Procedures

We conducted a cross‐sectional survey of PWID and MSM in India from November 2022 to May 2024. PWID were recruited in Amritsar, Ludhiana, Bilaspur, Churachandpur, New Delhi and Kanpur. MSM were recruited in Bhopal, New Delhi (separate from PWID site) and Hyderabad. Study enrolment was initiated on a rolling basis across cities with some, but not complete, overlap of enrolment periods. Participants were recruited using respondent‐driven sampling (RDS). Eligibility criteria included: (1) age ≥18 years; (2) oral informed consent; and (3) possession of a valid RDS referral coupon. PWID had to report injection drug use in the prior 2 years. MSM had to identify as male and report oral/anal sex with a man in the prior year.

Participants completed an interviewer‐administered survey in local languages and provided a blood sample. On‐site testing for HIV acquisition was conducted according to Indian guidelines [18].

### 2.2 Measures

Questions about LAI ART were asked of those with a prior HIV diagnosis. First, a short description was provided: “There are now new antiretroviral medicines that can be given by injection once every month to every two months that can treat your HIV infection. If you take these injections, you will not need to take daily ART tablets.” Then, acceptability was measured using a 4‐point Likert scale on the level of willingness “to take an injection to treat HIV infection” with options ranging from no chance to very good chance and undecided, don't know, and refused.

Awareness of PrEP (i.e. “Have you ever heard of pre‐exposure prophylaxis or PrEP to prevent HIV infection”) were asked of all participants. PrEP acceptability questions were restricted to those who did not report a prior HIV diagnosis. Immediately after the awareness question, acceptability was individually assessed for: daily oral pill, once monthly oral pill, implant placed every 6 months and LAI, with each noted as “reducing their risk of getting infected with HIV.” LAIs were described as “PrEP medicines given as injections once a month, once every 2 months, or once every 6 months” and an implant as “a tiny rod (thin as a needle and flexible) placed under your skin.” Participants were asked “Would you be willing to take [each modality] to reduce your risk of getting infected with HIV” with the same Likert responses used for LAI ART acceptability. After modality‐specific questions, participants ranked PrEP modalities in their order of preference, with an option to select that they would not take any PrEP options—an available option even if they indicated a willingness to use a PrEP modality in the prior section.

### 2.3 Statistical Analyses

Analyses were stratified by population. Descriptive analyses incorporated RDS‐II weights and a weight to account for differing population sizes within cities [19, 20]. LAI ART and PrEP acceptability was defined as reporting a very good chance that they would use injections for treatment/prevention. We assessed correlates of acceptability using Poisson regression models with robust variance to estimate prevalence ratios (PRs) and 95% confidence intervals, incorporating RDS‐II weights as probability weights. Age was included in multivariable models regardless of statistical significance; other correlates were retained if *p*‐value<0.05.

To understand PrEP preferences, we used a modified Borda count method [21] among those who responded they would take any PrEP modality. Modalities were ranked 1–4, summed across participants, incorporating weights, with lower sums indicating higher preference. Initial challenges with collecting PrEP rank order preference data from participants required question revision and resulted in missing data on this question from two sites—Bilaspur and New Delhi‐MSM. Eight MSM and five PWID were missing PrEP module responses and were excluded from PrEP analyses.

### 2.4 Ethical Clearances

This study was approved by the institutional review boards at Johns Hopkins University School of Medicine and YRGCARE (Chennai, India).

## 3 Results

### 3.1 Study Population

A total of 2249 MSM were recruited. Median age among MSM was 28 years, 45% were currently married, 71% had at least a high school education, median number of recent male/transgender partners in the prior 6 months was 10 and 47% reported condomless anal intercourse (CAI) in the prior 6 months; 20% were living with HIV. Overall, 13% (*n* = 224) of MSM reported a prior HIV diagnosis, of whom 90% were virally suppressed. Overall, 87% (*n* = 2025) did not have a prior diagnosis, of whom 48% reported recent CAI and 8% were living with undiagnosed HIV.

A total of 4499 PWID were recruited. Median age among PWID was 29 years, 2% were female, 39% currently married, 16% had at least a high school education, 29% had unstable housing in the prior year and 99% injected drugs in the prior 6 months, among whom 53% were injecting daily and 53% shared needles/syringes; 41% were living with HIV. Overall, 18% (*n* = 797) of PWID reported a prior HIV diagnosis; among those confirmed positive (*n* = 773), 42% were virally suppressed. Overall, 81% (*n* = 3702) did not have a prior diagnosis, of whom 34% recently shared needles/syringes, and 29% were living with undiagnosed HIV.

### 3.2 Acceptability of Long‐Acting Injectable ART

Among 224 MSM previously diagnosed with HIV, 89% reported a very good chance they would use LAI ART (Table 1). Correlates of higher LAI ART acceptability among MSM were identifying as *kothi* (adjusted PR [aPR] = 1.63), bisexual (aPR = 1.59), and other sexual identity (aPR = 1.93) and unstable housing (aPR = 1.22) (Table 2).

**TABLE 1:** Acceptability of long‐acting injectable antiretroviral therapy (LAI ART) and pre‐exposure prophylaxis (PrEP) modalities among men who have sex with men (MSM) and people who inject drugs (PWID) in India.

LAI ART acceptability among those who reported a prior HIV diagnosis		
n (%)	MSM (*N*=224)	PWID (*N*=797)
Acceptability of LAI ART		
No/very little chance	13 (2.0)	53 (4.1)
Some chance	29 (7.4)	134 (20.8)
Very good chance	177 (89.4)	598 (74.5)
Undecided/don't know	3 (0.3)	12 (0.6)
Refused to answer	2 (1.0)	0
Preferred frequency of LAI ART *(among those reporting some/very good chance)*		
Once a month	20 (21.8)	134 (16.1)
Once every 2 months	67 (25.4)	71 (14.4)
Once every 3 months	49 (29.5)	130 (26.8)
Once every 6 months	70 (23.3)	397 (42.7)

**PrEP acceptability by modality among those who without a prior HIV diagnosis**		
n (%)	**MSM (*N*=2020)**	**PWID (*N*=3694)**
Acceptability of daily oral pill		
No/very little chance	471 (11.3)	485 (9.1)
Some chance	350 (10.0)	910 (24.9)
Very good chance	1180 (78.3)	2274 (65.8)
Undecided/don't know	18 (0.3)	17 (0.2)
Refused to answer	1 (0.1)	8 (0.1)
Acceptability of monthly oral pill		
No/very little chance	456 (10.2)	421 (7.8)
Some chance	392 (12.9)	974 (32.4)
Very good chance	1152 (76.5)	2268 (59.0)
Undecided/don't know	20 (0.4)	22 (0.7)
Refused to answer	0	9 (0.1)
Acceptability of implant every 6 months		
No/very little chance	809 (15.4)	833 (16.8)
Some chance	405 (13.9)	11199 (35.3)
Very good chance	776 (70.0)	1606 (46.8)
Undecided/don't know	28 (0.7)	42 (0.8)
Refused to answer	2 (0.04)	14 (0.3)
Acceptability of long‐acting injectable (LAI PrEP)		
No/very little chance	482 (11.0)	418 (7.8)
Some chance	348 (11.6)	1152 (30.0)
Very good chance	1172 (76.8)	2098 (61.9)
Undecided/don't know	18 (0.5)	18 (0.2)
Refused to answer	0	8 (0.1)
Preferred frequency of LAI PrEP *(among those reporting some/very good chance)*		
Once a month	228 (31.8)	665 (17.6)
Once every 2 months	119 (12.3)	568 (22.4)
Once every 3 months	263 (24.1)	620 (27.7)
Once every 6 months	910 (31.8)	1397 (32.4)

*Note*: Cities represented in data include: MSM—Bhopal, New Delhi and Hyderabad; PWID—Amritsar, Ludhiana, Bilaspur, Churachandpur, New Delhi and Kanpur. Percentages are weighted using RDS‐II weights and population size weights.

Abbreviation: RDS, respondent‐driven sampling.

**TABLE 2:** Correlates of long‐acting injectable antiretroviral therapy (LAI ART) and long‐acting injectable pre‐exposure prophylaxis (LAI PrEP) acceptability among men who have sex with men (MSM) and people who inject drugs (PWID) in India.

	LAI ART acceptability among those who reported a prior HIV diagnosis		LAI PrEP acceptability among those who without a prior HIV diagnosis	
	Adjusted PR (95% CI) MSM (*N*=224)	Adjusted PR (95% CI) PWID (*N* = 797)	Adjusted PR (95% CI) MSM (*N*=2020)	Adjusted PR (95% CI) PWID (*N* = 3694)
**Socio‐demographics**				
Age (years)				
18−25	Reference	Reference	Reference	Reference
26−35	1.02 (0.94, 1.11)	0.99 (0.86, 1.15)	0.94 (0.88, 1.01)	1.02 (0.93, 1.11)
36−45	0.86 (0.64, 1.16)	0.93 (0.78, 1.12)	0.96 (0.88, 1.04)	1.12 (1.00, 1.26)
>46	0.86 (0.72, 1.03)	0.91 (0.73, 1.13)	0.87 (0.72, 1.05)	1.29 (1.11, 1.49)
Sexual identity *(MSM)* ^a^				
*Panthi*	Reference		Reference	
*Kothi*	1.63 (1.11, 2.40)		1.59 (1.19, 2.12)	
Double decker	1.34 (0.71, 2.53)		1.87 (1.31, 2.69)	
Gay/MSM	1.44 (0.96, 2.17)	−	2.32 (1.79, 3.01)	−
Bisexual	1.59 (1.08, 2.34)		2.33 (1.80, 3.01)	
Other	1.93 (1.24, 3.01)		0.95 (0.30, 2.99)	
Currently married	−	−	−	0.84 (0.77, 0.92)
High school education and above	−	−	−	0.89 (0.80, 0.99)
Wage pattern				
Monthly/weekly			Reference	
Daily/seasonal	−		0.83 (0.64, 1.07)	
Unemployed/laid off		−	0.89 (0.68, 1.15)	−
Other (retired, student, homemaker, etc.)			0.87 (0.77, 0.98)	
Unstable housing in prior 12 months	1.20 (1.03, 1.41)	−	−	−
Moderately severe or severe depression^b^	−	−	−	0.86 (0.79, 0.93)
Takes medication daily or most days	−	−	—	0.78 (0.64, 0.97)
**Sexual risk behaviours and sexually transmitted infections**				
Number of male/transgender sexual partners in the prior 6 months *(MSM)*				
None			Reference	
1			1.72 (1.28, 2.31)	
2−5	—	—	1.79 (1.36, 2.36)	—
6−15			1.91 (1.47, 2.48)	
16 or more			1.94 (1.49, 2.53)	
Number of sexual partners of any gender in prior 6 months				
None				Reference
1				1.34 (1.23, 1.46)
2 or more *(PWID)*/2−5 *(MSM)*	—	—	—	1.35 (1.17, 1.56)
6−15 *(MSM)*				
16 or more *(MSM)*				
Condomless anal intercourse in prior 6 months *(MSM)*	—	—	0.90 (0.85, 0.96)	—
Sexually transmitted infection symptoms (anal/rectal discharge, pain or sore/ulcer) in prior 6 months	—	—	1.09 (1.01, 1.17)	1.16 (1.01, 1.34)
**Substance use and risk behaviours**				
Medication for opioid use disorder use *(PWID)*				
Never		Reference		Reference
Previously but not in prior 6 months	—	1.01 (0.90, 1.14)		0.93 (0.84, 1.04)
In prior 6 months		0.69 (0.53, 0.89)	—	0.70 (0.58, 0.84)
Heavy or dependent alcohol use^c^	—	—	1.08 (1.02, 1.13)	0.79 (0.68, 0.91)
**HIV**				
HIV viral load <150 copies/mL^d^ *(among those with confirmed HIV infection)*	—	1.16 (1.03, 1.30)	—	—
Heard of PrEP before	—	—	—	1.31 (1.18, 1.45)
Living with undiagnosed HIV	—	—	—	1.13 (1.04, 1.22)

*Note*: Cities represented in data include: MSM—Bhopal, New Delhi and Hyderabad; PWID—Amritsar, Ludhiana, Bilaspur, Churachandpur, New Delhi and Kanpur. Poisson models incorporate RDS‐II weights as probability weights.

Abbreviations: CI, confidence interval; PR, prevalence ratio; RDS, respondent‐driven sampling.

^a^ *Panthi*—masculine appearance and primarily insertive anal sex; *kothi*—feminine appearance and primarily receptive anal sex; *Double Decker*—both insertive and receptive anal sex.

^b^ Assessed using the PHQ‐9 (score≥10) (Kroenke K, Spitzer R, Williams J. The patient health questionnaire (PHQ‐9)–overview. *J Gen Intern Med*. 2001;16:606‐616. See reference #30).

^c^ Assessed using the AUDIT‐C (score≥4 for men, ≥3 for women) (Bush K, Kivlahan DR, McDonell MB, Fihn SD, Bradley KA. The AUDIT alcohol consumption questions (AUDIT‐C): an effective brief screening test for problem drinking. Ambulatory Care Quality Improvement Project (ACQUIP). Alcohol Use Disorders Identification Test. *Arch Intern Med*. Sep 14 1998;158(16):1789‐1795. See reference #31).

^d^ Plasma HIV‐1 viral load was measured using RealTime HIV‐1 assay (Abbott Park, IL, USA).

Among 797 PWID previously diagnosed with HIV, 75% reported a very good chance they would use LAI ART (Table 1). Viral suppression (aPR = 1.16) was associated with higher LAI ART acceptability. Recent medication for opioid use disorder (MOUD) was associated with lower LAI ART acceptability (aPR = 0.69) (Table 2).

### 3.3 Awareness and Acceptability of PrEP

Among all MSM, 20% reported ever hearing of PrEP to prevent HIV acquisition. Among 2020 MSM without a prior HIV diagnosis, 78% reported a very good chance they would use daily oral pills, 77% for monthly oral pills, 70% for an implant and 77% for LAI (Table 1). In Hyderabad, 61–73% reported no/very little chance for all PrEP modalities, while in Delhi, 89–93% reported a very good chance. Correlates of higher LAI PrEP acceptability among MSM were identifying as *kothi* (aPR = 1.59), double decker (aPR = 1.87), gay/MSM (aPR = 2.33), and bisexual (aPR = 2.33), ≥1 recent male/transgender sexual partner, sexually transmitted infection (STI) symptoms (aPR = 1.09) and heavy‐dependent alcohol use (aPR = 1.08) (Table 2). Another wage pattern (aPR = 0.87)—largely representing a student status—and CAI (aPR = 0.90) were associated with lower LAI PrEP acceptability.

Among all PWID, 5% reported ever hearing of PrEP to prevent HIV acquisition. Among 3694 PWID without a prior HIV diagnosis, 66% reported a very good chance they would use daily oral pills, 59% for monthly oral pills, 46% for an implant and 62% for LAI (Table 1). Bilaspur and Churachandpur reported a higher percentage (24–52%) with no/very little chance across modalities. Correlates of higher LAI PrEP acceptability among PWID were age ≥46 years (aPR = 1.29), ≥1 recent sexual partner, STI symptoms (aPR = 1.16), hearing of PrEP before (aPR = 1.31) and undiagnosed HIV (aPR = 1.13) (Table 2). Being married (aPR = 0.85), ≥high school education (aPR = 0.89), moderately severe‐severe depression (aPR = 0.86), taking regular medications (aPR = 0.78) and recent MOUD (aPR = 0.70) were associated with lower LAI PrEP acceptability.

### 3.4 Preferred PrEP Modalities

**FIGURE 1:**
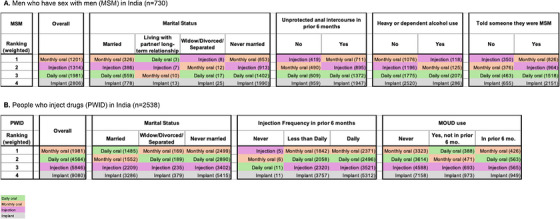
Borda count ranking of pre‐exposure prophylaxis (PrEP) modalities. (A) Men who have sex with men (MSM) in India (*n* = 730). (B) People who inject drugs (PWID) in India (*n* = 2538). Numbers in parentheses are the ranking sum for each modality with the most preferred assigned a value of 1, second preferred a value of 2, and so on. Sums incorporated RDS‐II and population size weights. Cities represented in data include: MSM – Bhopal, Hyderabad; PWID – Amritsar, Ludhiana, Churchandpur, New Delhi, Kanpur. PrEP ranking data from New Delhi (MSM) and Bilaspur were omitted due to initial data collection challenges.

Among MSM without a prior HIV diagnosis (excluding New Delhi), 55% (*n* = 730) would take PrEP with monthly oral pills most preferred, followed by LAI, daily oral pills and then implant (Figure 1A). Preference order was consistent across subgroups except those who did not have recent CAI, with heavy/dependent alcohol use, and who had never told someone they were MSM, for whom LAI was most preferred, followed by monthly oral pills.

Among PWID without a prior HIV diagnosis (excluding Bilaspur), 79% (*n* = 2538) would take PrEP with monthly oral pills most preferred, followed by daily oral pills, LAI i and then implant (Figure 1B). Preference order was consistent across subgroups except those married and those with a prior history of MOUD, for whom daily oral pills was most preferred, followed by monthly oral pills.

## 4 Discussion

Among community‐based MSM and PWID in India, there was high acceptability of LAI ART and all PrEP modalities—LAI, daily oral, monthly oral and implant. Despite high PrEP acceptability, the majority had not heard of PrEP before. When asked about PrEP preferences, 45% of MSM and 21% of PWID were not interested in taking any PrEP options. This low PrEP interest among MSM is partially explained by city‐level variability; MSM in New Delhi were most enthusiastic about individual PrEP modalities but were excluded from preference analyses. Among those interested in PrEP, most would prefer a monthly oral pill—such as MK‐8527 under investigation [22]—over LAI PrEP.

Oral PrEP, while approved for use in India, is not available from the public sector—consequently, there is low awareness and uptake of PrEP (∼7000 cumulative initiations [23]). These findings should be interpreted in light of the current context and estimates of acceptability and interest considered as a starting place for designing and implementing future services for these populations. Notably, a predictor of LAI PrEP acceptability among PWID was prior PrEP knowledge. Person‐focused and tailored dissemination and educational strategies would likely increase acceptability and demand for these medications.

We found there was more willingness to take LAI ART compared to LAI PrEP. PWH may value the benefit of infrequent injections over daily oral pills after their prior treatment experiences. Indeed, a predictor of LAI ART acceptability among PWID was current viral suppression, suggesting those who successfully treat their HIV with daily oral medication were most enthusiastic about LAI ART, likely due to the inconveniences of daily pills alongside a commitment to ART adherence.

When PrEP options were ranked, a monthly oral pill was most preferred among both population groups, with LAI PrEP ranking second among MSM and third among PWID after daily oral pills. Studies among MSM in the United States have found that oral PrEP modalities are often preferred over injectable, especially event‐based prevention methods [24, 25]. In our study, MSM who reported recent CAI were less willing to use LAI PrEP, indicating a group for whom on‐demand pills could be appealing and effective. Prior work in India has found interest in this strategy among MSM [26, 27]. For PWID, we found a preference for daily oral PrEP over LAI PrEP [28]. There were similar findings among PWID in the San Diego‐Tijuana area, where 44% were only interested in daily oral PrEP, while 25% were only interested in LAI PrEP [29].

Our study included data from multiple diverse cities across India and community‐based samples of MSM and PWID. However, results may not be transportable to other settings, and prior PrEP use was not assessed.

## 5 Conclusions

In conclusion, MSM and PWID enrolled in our study in India were interested in LAI formulations for HIV treatment and prevention. Once these formulations become available for use in India, re‐assessing awareness and acceptability, as well as community outreach and education, will be critical to success. For PrEP, continued investment in the development of extended‐duration oral formulations is warranted and valuable [30, 31].

## Author Contributions

AMM, AKS, SSS, SHM and GML—study concept, design, data interpretation. JJB, AK and AS—data acquisition and interpretation; TAL—data analysis and interpretation. All authors have read and approved the final manuscript.

## Funding

The study was funded by the National Institutes of Health: R01DA041034, K24DA035684, T32AI102623 and DP1DA060602.

## Conflicts of Interest

SHM has received materials support from Abbott Diagnostics. SSS serves as the Managing Trustee of the YR Gaitonde Medical Education Research Foundation and on the Board of Directors of the Serious Fun Children's Network. SSS has also received grants, products and honoraria from Abbott Laboratories and Gilead Sciences, not related to the work presented in the manuscript. All other authors declare that they have no competing interests.

## Data Availability

The data that support the findings of this study are available from the corresponding author upon reasonable request.
